# Statistical Properties and Robustness of Biological Controller-Target Networks

**DOI:** 10.1371/journal.pone.0029374

**Published:** 2012-01-03

**Authors:** Jacob D. Feala, Jorge Cortes, Phillip M. Duxbury, Andrew D. McCulloch, Carlo Piermarocchi, Giovanni Paternostro

**Affiliations:** 1 Sanford-Burnham Medical Research Institute, La Jolla, California, United States of America; 2 Department of Mechanical and Aerospace Engineering, University of California San Diego, La Jolla, California, United States of America; 3 Department of Physics and Astronomy, Michigan State University, East Lansing, Michigan, United States of America; 4 Department of Bioengineering, University of California San Diego, La Jolla, California, United States of America; Mount Sinai School of Medicine, United States of America

## Abstract

Cells are regulated by networks of *controllers* having many targets, and *targets* affected by many controllers, in a “*many-to-many*” control structure. Here we study several of these bipartite (two-layer) networks. We analyze both naturally occurring biological networks (composed of transcription factors controlling genes, microRNAs controlling mRNA transcripts, and protein kinases controlling protein substrates) and a drug-target network composed of kinase inhibitors and of their kinase targets. Certain statistical properties of these biological bipartite structures seem universal across systems and species, suggesting the existence of common control strategies in biology. The number of controllers is ∼8% of targets and the density of links is 2.5%±1.2%. Links per node are predominantly exponentially distributed. We explain the conservation of the mean number of incoming links per target using a mathematical model of control networks, which also indicates that the “*many-to-many*” structure of biological control has properties of efficient robustness. The drug-target network has many statistical properties similar to the biological networks and we show that drug-target networks with biomimetic features can be obtained. These findings suggest a completely new approach to pharmacological control of biological systems. Molecular tools, such as kinase inhibitors, are now available to test if therapeutic combinations may benefit from being designed with biomimetic properties, such as “*many-to-many*” targeting, very wide coverage of the target set, and redundancy of incoming links per target.

## Introduction

**Figure 1:**
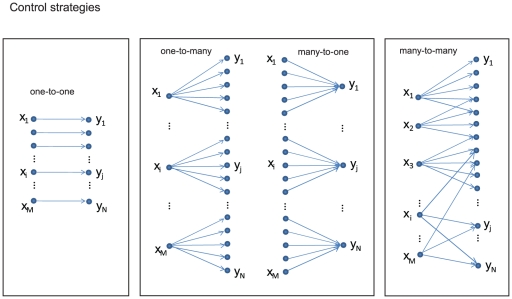
Possible combinatorial control strategies. There are several qualitatively different structures for control networks of M controllers (x_1_,x_2_,…x_M_) and N targets (y_1_,y_2_,…y_N_). In the one-to-one case (left panel), M = N.

Control of cellular function depends on bipartite (two-layer) networks, in which one class of nodes (the controller) acts on the other class (the target) to regulate its function. Examples of cellular control networks include transcription factors, microRNAs, and protein kinases, which control genes, mRNA transcripts, and protein substrates, respectively. In these networks, the control layer interacts with the target layer in a combinatorial, “many-to-many” fashion (see Figure 1). In other words, each controller has many targets, the targets themselves are under the influence of many controlling molecules, and the target sets of different controllers overlap. Moreover, the number of controllers is usually significantly lower than the number of targets. This “many-to-many” structure is well recognized in biological systems [1], not only in intracellular control but also in many other types of complex control in biology, including the nervous system (see Text S1, section S1.1).

The idea of a many-to-many bipartite control structure is similar to the concept of dense overlapping regulon (DOR) [2] in bacterial gene networks, which indicates a motif (i.e. a pattern that recurs within a network), in which transcription factors and genes are connected through many overlapping interactions. Here we extend this concept to different biological structures and describe the many-to-many property as a feature of entire control networks, for different types of control molecules, contrasting it with the other possible bipartite structures, such as one-to-one and one-to-many, described in Figure 1. One important question concerns the statistical properties of these control structures with strong overlap and redundancy. It was shown [2] that dense overlapping regulons deviate substantially from random networks. Here we explicitly characterize the global statistical properties of several bipartite control structures, and we show that the degree distribution of the two types of nodes is well approximated by exponentials.

A key issue related to network topology is robustness. What are the advantages of the “many-to-many” structure in terms of robustness, and why, as we show here, do some parameters of the networks seem to be universal across different control structures and species? In order to explore the link between the network properties and robustness we introduce a simplified Boolean signaling model. Boolean network models of biological regulation were first pioneered by Kaufmann [3] [4], and have been used to model specific interactions in small, well-characterized biological pathways [5], [6], [7]. The control problem – i.e. calculating the specific input sequence required to achieve a desired output – has also been explored within these systems [8], [9]. None of these models explicitly considered bipartite structures, i.e. networks with two classes of nodes in which there are no links between nodes of the same class. While there have been many genome-wide network analyses [10], [11], [12], [13], [14], [15], and one recent work on co-regulation of transcription and phosphorylation networks [16], here we focus exclusively on universal features of bipartite networks, neglecting the fact that some of the targets might also act in turn as controllers on other downstream biological entities or on other targets. This simplified approach captures some peculiar and universal properties of control in biology that may help design biomimetic drug-target control strategies.

## Results

### Naturally occurring biological control networks share statistical properties

We examine quantitative characteristics of three biological control systems in three different species (human, yeast, and *E. coli*), from the perspective of bipartite combinatorial control. First we consider the numbers of nodes. Table 1 (upper left) shows estimates of the number of controllers and targets from the literature for the three types of networks in humans. Notably, though these numbers are from three different cellular systems of varying size, the ratios of control nodes to target nodes are similar, approximately 8% (Table 1, upper left). We also measured the controller/target ratio in several molecular interaction databases. These databases are sparse and therefore provide less confident estimates than the literature, but we found a similar mean value: 8.9% (albeit with much higher variability).

**Table 1:** Network parameters for various types of combinatorial control within cells.

	Literature			Network databases						
	Human			Human			Yeast		E. coli	Drug
*Node properties*	*TF*	*Kinase*	*miRNA*	*TF*	*Kinase*	*miRNA*	*TF*	*Kinase*	*TF*	*KI*
Controllers (M)	1,800*	518	940	389	264	153	186	88	169	38
Targets (N)	20,500•	6,150‡	11,890†	9284	988	9448	6297	1341	1495	316
M/N (%)	8.8%	8.4%	7.9%	4.2%	26.7%	1.6%	3.0%	6.6%	11.3%	12.0%
***Link properties***										
Outgoing links from controllers (mean k_out_)				181	8.9	359	229	46	20	78.8
Incoming links per target (mean k_in_)				7.6	2.4	5.8	6.8	3	2.3	9.48
Link density				1.9%	0.9%	3.5%	3.6%	3.5%	1.3%	25.0%
Shared targets per controller (mean)				98%	73%	95%	98%	85%	74%	100%
Pairwise overlap of targets (mean)				4.5%	7.1%	7.1%	6.3%	8.3%	1.1%	33.8%

Statistical values of selected parameters					
Parameter	Mean	SD	CV	95% lo	95% hi
M/N (literature)	8.4%	0.5%	0.054	7.5%	9.3%
Incoming links per target (k_in_)	4.7	2.4	0.51	0.016	9.3
Link density	2.5%	1.2%	0.50	0.041%	4.9%
Shared targets per controller	87.2%	11.6%	0.13		
Pairwise overlap of targets	5.7%	2.6%	0.45	0.6%	10.8%

*Vaqueriza et al. [51] estimate 1,700–1,800 human transcription factors.

• Other estimates for the number of human genes are in the range 20,000–25,000.

† Friedman et al. [40] estimate 58% of genes are targeted by miRNA (11,890 = .58*20,500).

‡ Cohen et al. [52] estimate 30% of human proteins are phosphoryated (6,150 = .30*20,500).

The ratio of controllers per target drawn from the literature is similar across different types of biological network in humans, approximately 8%. Node properties differ between the literature and network databases owing to incomplete information in the databases. Link density is the ratio of the number of actual links to the number of possible links. Shared targets per controller and pairwise overlap are measurements of overlapping target sets described in the Text S1, section S1.3. SD = standard deviation, CV = coefficient of variation.

Next, we use molecular interaction databases to explore connectivity parameters of bipartite networks in nature. Networks were extracted from publicly available databases and separated into controller nodes (microRNA, transcription factors, protein kinases) and target nodes (mRNA transcript, gene, protein substrates), with directed links between controllers and targets. We quantified properties including density of links (existing links divided by the number of possible links), distribution of links for each type of node, and overlap between the target sets of different controllers. In these datasets, the percentage of targets that also act as controllers is very small and sizeable only in the human transcription factor network (1.6%) and in the human kinase network (16%) (see Text S1, section S1.2 and Table S1 for more details).

Table 1 shows that these networks share specific network-wide properties despite wide variation in the number of nodes, complexity of species, and type of molecular interaction. As mentioned above, the mean controllers per target (M/N) over all biological networks was 8.9%. Detailed analysis of Gene Ontology (GO) enrichment of targets is described in Text S1 section S1.2, Figure S1, and Tables S2 and S3. Analyses of the two measures of overlap (Shared Targets per Controller and Pairwise Overlap of Targets, see Figure S2 for an illustrative definition) are described in Text S1 section S1.3 and Table S4.

We have observed that the networks in the databases are all characterized by the presence of a giant connected component. In particular, the human and yeast transcription factor, human miRNA, and kinase inhibitors networks are completely connected. Human and yeast kinase networks contain a few disconnected components with two and three nodes. Only in the E. coli transcription factor network there is a considerable fraction of nodes (7%) outside the giant component. These nodes are grouped in many disconnected small components of size ranging from 2 to 11.

**Figure 2:**
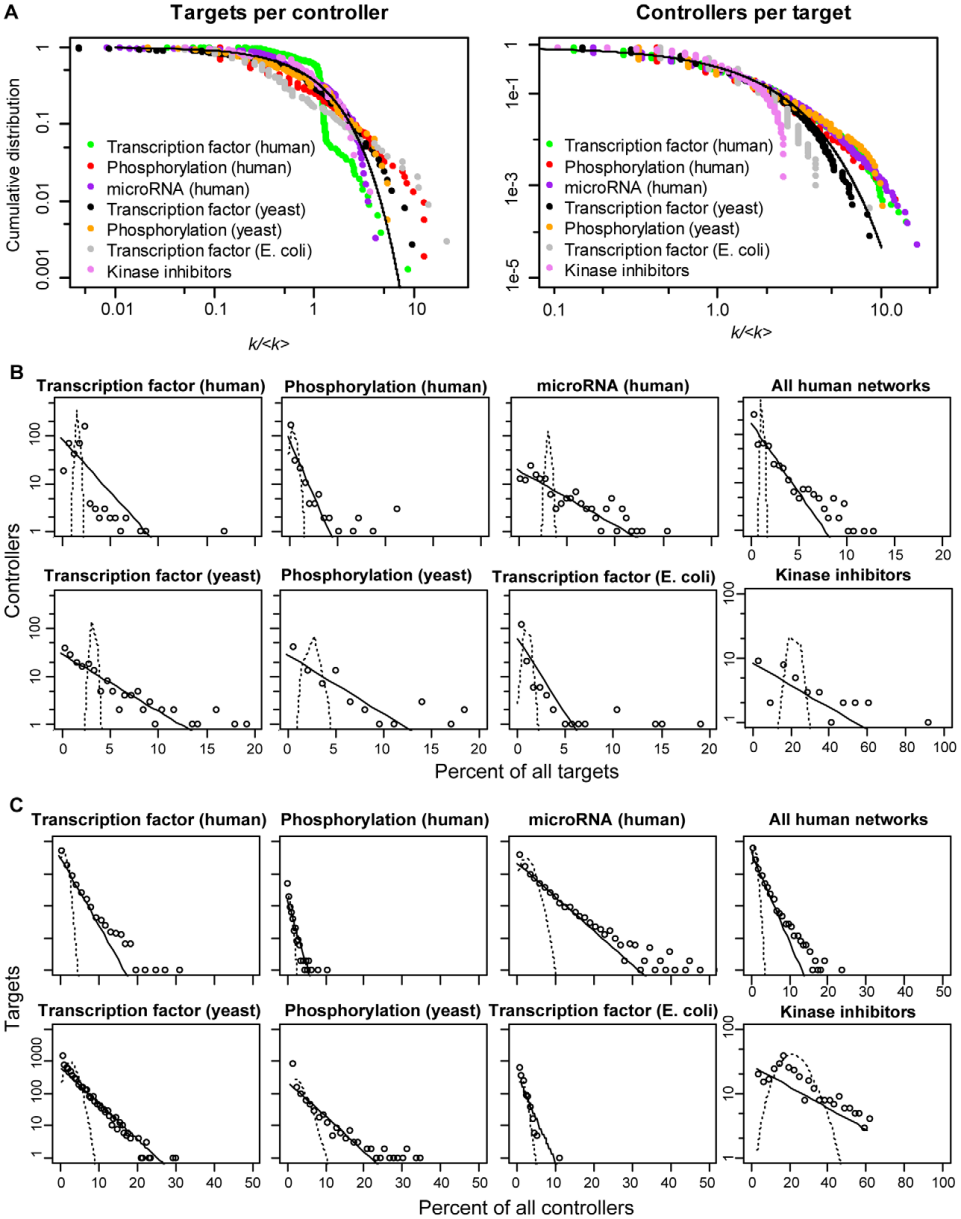
Distributions of incoming and outgoing links for several types of combinatorial control networks. (A) Cumulative distributions of links per node in each of the networks of Table 1 were normalized by the mean and plotted together on log-log axes, alongside the discrete analog to the exponential distribution (solid line), see Methods. By contrast, a power-law, or scale-free, distribution would produce a straight line in this log-log plot. (B) Individual histograms of targets per controller (outgoing links from controllers, *k_out_*), and (C) controllers per target (incoming links per target, *k_in_*) plotted for each individual network. The three human networks were combined based on shared targets (top right of each panel). Horizontal axes in (B) and (C) are normalized to the total number of target or controller nodes, respectively in each network. Each distribution is compared with the binomial distribution expected from a bipartite random graph with identical numbers of nodes and links (dashed curve). An exponential curve is also fitted to each dataset (solid line). Note that the kinase inhibitor network shown here is distributed over a much wider range on the x-axis than the biological networks.

We explored properties of the node degree distributions in the bipartite networks. Figure 2 shows distributions of links per node *k*, for incoming links per target (controllers per target, *k_in_*) and outgoing links from controllers (targets per controller, *k_out_*). Figure 2A depicts the empirical cumulative distribution function (cdf) for all datasets, normalized by the average links per node *<k>* and overlaid on a standard exponential cdf (solid line). Figures 2B and 2C compare histograms of each network with bipartite random networks of the same size (a modified Erdös-Rényi random graph model in which edges between controllers and targets are generated with constant probability, see Methods). Only the human transcription factor network has a peak in its outgoing link distribution that is compatible with the binomial distribution characteristic of bipartite random graphs. The incoming links in the kinase inhibitor network also show a possible binomial component. Otherwise, most curves approximate an exponential distribution, which is not consistent with a bipartite random graph model (further analyses of curve-fitting and link distributions are provided in Text S1 sections S1.4, S1.6, Figures S3, S4, S5, S6, and S7, and Table S5).

Notably, the average <*k_in_*> of targets lies within the narrow range between 2 and 10 for all networks studied, a phenomenon which we explore below in more detail using a mathematical model. These global averages cannot be statistically tested against a degree-preserving null model, however, as the randomized networks would have exactly the same average values of incoming and outgoing links as the test network. We instead used degree-preserving randomization to test correlations between *k_out_* and *k_in_* for each network (see Text S1 section S1.5 and Figure S8), following the method described by Maslov et al. [17]. Though these in-degree/out-degree correlation patterns were not found to be as robustly conserved as other statistical properties, the analysis reveals trends that may be interesting avenues for future research.

All biological networks had similar sparse link density, realizing an average of only 2.5%±1.2% of all possible controller-to-target interactions. Link density *D* is related to the average links per node by the equation [18]

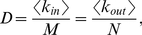
(1)where *<k_in_>* is the average incoming links over *N* target nodes, and *<k_out_>* is the average outgoing links from *M* controller nodes. Note that
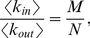
(2)suggesting that similarities in the ratios of nodes may be related to constraints on the average incoming and outgoing links per node.

### A drug-target network with biomimetic properties can be sampled from a large drug library

We also analyzed a drug target network composed of 38 kinase inhibitors and of their kinase targets [19]. This network has also a many-to-many structure and its properties have similarities but are not identical to the biological ones (see Table 1 and Figure 2).

This published drug-target dataset was a small sample, however, compared to existing libraries of thousands of fully profiled (i.e., with known targets) kinase inhibitors owned by pharmaceutical or biotech companies. Information about the size of these profiled libraries can be found in some official documents (e.g, see Ambit IPO S-1 SEC 2010 filing). In the absence of drug-target data from these proprietary libraries, we therefore simulated a kinase inhibitor library of a comparable size. We simulated the drug-target network for a hypothetical library of 1500 compounds, creating target profiles that gave the same target per controller and controller per target distributions as the 38-drug network in Karaman et al. [19]. We used the simulated network to show that, by sampling existing drug libraries, it is possible to identify sets of kinase inhibitors with statistical properties very similar to those of biological controllers.

The simulated library was created using the inverse sampling transform method, which requires the analytic inversion of the cumulative distributions of the theoretical distributions we want to sample [20]. This method is used both for targets and for controllers. A link-matching procedure is then implemented to randomly match “links out” of kinase inhibitors with “links in” into kinase nodes, creating a bipartite network with the desired link distributions. We show in Figure S9 the outgoing links from controllers and incoming links per target for a simulated network obtained with this procedure.

Once a sample kinase inhibitor/kinase network has been created, we have used a rejection method approach [20] to identify a subset of inhibitors having an exponential distribution, but a reduced average value for *<k_out_>*, more similar to our measurements in the naturally occurring networks. The rejection method consists in picking randomly an inhibitor node with a *k_out_* = *k*, and keeping it in the set with probability 
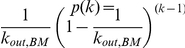
, where *k_out,BM_* is the ideal biomimetic value. In implementations using a real drug library, biological information about the targets can be incorporated, using a modified alternative of the sampling algorithm (see Methods for details).

The simulated library (see also Figure S9) is composed of 1,500 kinase inhibitors targeting all the 518 kinases in the human genome. In this larger library the average *k_out_* was 55 and the average *k_in_* was 159. The smaller sampled library composed of 60 kinase inhibitors targeting 486 kinases (a coverage of 93.8% of all kinases). In this library the average *k_out_* was 43 and the average *k_in_* was 5.3. The statistical parameters of the sampled library are closer to the naturally occurring ones shown in Table 1.

### A Boolean bipartite model shows dependence of robustness on <k_in_>

The many-to-many network structure, with parameters spanning comparatively limited ranges, may be the result of an optimized trade-off between efficient use of biological resources and robustness (via redundancy) to variation in environmental and genetic inputs. To maximize redundancy, a high average incoming link per target value is clearly preferable. We built a model to simulate redundancy and robustness in a bipartite signaling network. A set of transcription factors, for example, takes on its expression state according to upstream signaling events, and induces an output gene expression state through its network of targets. Now consider a set of *M* controller nodes, which can take on 2^M^ binary states. Controllers are randomly connected to *N* target nodes having average incoming links *<k_in_>*, and each target node takes on a binary state according to a Boolean rule on unweighted links (see Methods). We can then derive the number of unique output sequences *Ω* that the network can achieve, and the robustness *R* of an output state to mutations (link deletions), given values of *M*, *N*, and *<k_in_>*.

**Figure 3:**
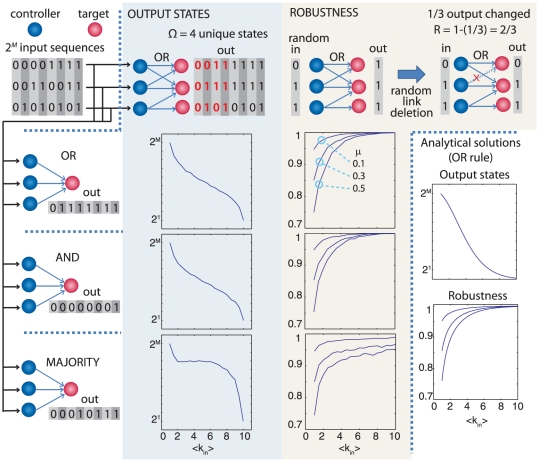
Mathematical model of the number and robustness of output states in a bipartite control network. We explored the dependence of these quantities on the average incoming links per target *<k_in_>*, number of controllers *M*, number of targets *N*, and mutation rate 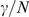 (or links deleted as a fraction of *N*, robustness equation only). Shown are averages of 1000 numerical simulations with *M* = *N* = 10, and 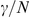 = 0.1. Analytical solutions for robustness and unique output states using the OR rule were also derived and plotted (lower right), and found to be identical or a close approximation to simulations, respectively (see Methods). Both quantities were independent of *N* in numerical and analytical solutions. These results suggest that marginal utility to robustness of increasing *<k_in_>* shrinks rapidly above ∼5, while at the same time incurring a cost on the degree of freedom of output states.

In Figure 3, analytical solutions for *Ω* and *R* are plotted as a function of *<k_in_>* over the biological ranges of Table 1, alongside numerical simulations (see Methods). Numerical results were similar regardless of whether the OR, AND, or MAJORITY rules were used, and analytical derivations for the AND and OR rule were equivalent by symmetry. The MAJORITY rule may be biologically relevant in some cases, but this rule is mathematically more complex. Therefore, the MAJORITY rule was simulated numerically but not derived analytically. Numerical simulations were intractable for large N, preventing us from simulating biological values of N or cases where M<<N. Numerical results are expected to approach the analytical curves at large N, however. Additionally, these equations are not dependent on N, and therefore incorporate the case M<<N as well.

The number of unique output states *Ω* is a decreasing function of *<k_in_>*, and robustness *R* is an increasing function of *<k_in_>* dependent on the mutation rate. Furthermore, *R* increases rapidly with *<k_in_>* above 1, but saturates quickly for values above 5. Therefore, adding redundancy via *<k_in_>* has a high marginal benefit to robustness for low *<k_in_>*, but as *<k_in_>* increases, the incremental benefit to *R* may be outweighed by the cost to the unique outputs achievable by the network. Marginal utility to robustness of increasing *<k_in_>* shrinks rapidly above ∼5, while at the same time incurring a cost on the number of feasible unique output states. This *<k_in_>* value is close to the naturally occurring values shown in Table 1.

## Discussion

### Trade-offs between robustness and efficiency

In addition to the quantitative conclusions of the Boolean model, other trade-offs might also be involved in determining the values of the observed parameters. There may be an additional evolutionary cost for attaining and storing the genetic information required for each link, and increasing the numbers of controllers and links may also incur a cellular cost for resources dedicated to protein synthesis. Many-to-many configurations would therefore be expected to emerge as a strategy for maximizing both robustness and the efficient use of resources, and observed network parameters reflect a balance between these opposing influences. These considerations are consistent with the differences in values of <*k_in_*> among human and bacterial transcription factor networks (Table 1). As pointed out by r/K selection theory [21], these two organisms use very different life history strategies, with bacteria favoring more rapid reproduction (facilitated by a smaller genome size) and lower offspring robustness.

### Biological networks and mathematical models of robustness

Robustness is a key feature of biological systems [22] and has been shown in different types of mathematical models of biological networks. Among these are Boolean network models, first pioneered by Kaufmann [3] [4]. Boolean rules have been used to model specific interactions in small, well-characterized biological pathways [5], [6], [7], and entropy-based methods have been used to examine the robustness and flexibility of a small pathway to achieve functional outputs [23].

Buldyrev et al [24] have presented a model of the vulnerability of interdependent networks. Interestingly, nodes from the two interdependent networks were connected among each other only by one-to-one links, providing additional evidence for the lack of robustness of this type of structure.

Besides structural robustness to the removal of nodes or links, several authors have also investigated dynamical robustness. For example, within a framework of dynamical modeling based on attractors, Li et al [6] have shown that the cell-cycle network is extremely stable and robust for its function. The robustness of dynamical networks with different degree distributions has been analyzed in terms of the presence or absence of attractor states also by other authors [25]. The robustness is given by the tendency of the system to return to the attractor states after perturbation. Klemm and Bornholdt [26] have investigated the reliability of information processing in networks of noisy switches with fluctuating response times. It would be informative to investigate in future work the behavior of the control structures and parameters we have described in this paper within these different dynamical models.

In contrast to previous studies, in this paper we analyze the properties of bipartite networks in terms of the allowed configurations that can be realized in the target nodes for all input states. The dynamics are therefore limited to a single step. Also in contrast to other studies, here we consider robustness in terms of how the number of accessible states is reduced by deleting links. We use this entropy of target states as a tool to examine the general parameter dependence of robustness and flexibility of Boolean control in bipartite networks of arbitrary size. Specifically we examine the dependence of robustness and flexibility on k_in_, one of the parameters shown to be conserved in our statistical analysis of biological networks.

### Enrichment of gene categories in network targets

As shown in more detail in Text S1.2, we used the three human networks to explore whether certain categories of nodes may be more highly targeted than others. Controller nodes appeared in the target sets more than expected (Table S1). Highly targeted genes in all networks shared many significantly enriched Gene Ontology (GO) terms [27] involved in transcription, regulation, and development (Table S2). Conversely, sparsely targeted genes tended to be enriched in GO terms involving biological “effector” processes, such as metabolism, transport, and the response to stimulus (Table S3). Additionally, human genes regulated by all three types of controller molecule were almost always themselves involved in regulation (Figure S1). Together these data suggest that cells use different control network topologies depending on the type of target genes. Control nodes themselves are under the heaviest combinatorial control, and by more different types of controller, while downstream effector genes are regulated by fewer controllers. These observations might be relevant to the design of strategies for pharmacological combinatorial control.

### Implications of the existence of biomimetic drug-target bipartite networks

Our results show that pharmacological sets with biomimetic statistical properties can be built from kinase inhibitor libraries available now in companies and this paper intends to provide a theoretical justification for experiments to test the effectiveness of this biomimetic approach to pharmacology.

The evolutionarily conservation of the many-to-many structure and of the statistical parameters and the results of our mathematical model suggests that pharmacological control strategies should be designed similarly. Current efforts to develop specific, targeted therapies follow the one-to-one approach to drug therapy [28], [29]; in other words, the ideal aim of drug discovery is seen as having one drug for each molecular target, with no target overlap. More traditional therapies are often less specific (one-to-many in Figure 1) and some effective targeted therapies have also been found to be non-specific and might fit this category [30], [31].

An alternative approach would seek combinations of drugs that control the activation state of a large proportion of a set of targets in a many-to-many fashion, similar to combinatorial regulation of cellular networks, rather than intervening at a single or small number of targets. Combinatorial therapies could be found by searching within biomimetic pharmacological sets having the same network structure as naturally occurring biological systems. Evolution conducts a similar search using all controller molecules encoded in the genome, in order to find the optimal subsets to be expressed in a particular cell type. The many-to-many approach may be more robust to drug resistance and to genetic and environmental variation, as suggested by our mathematical model.

There are two recent developments that make testing this approach a realistic possibility. The first is the emergence of high-throughput *in vitro* or *in vivo* search algorithms for efficiently optimizing large combinations of drugs from within candidate sets [32], [33], [34], [35]. These algorithms are essential to overcome the exponentially growing possibilities of the combinatorial space. It is clearly not sufficient for pharmacological sets to have an optimal network control structure, and these methods permit an efficient search for the appropriate component drugs. The second is the availability of large libraries of suitable molecular tools, the most promising being kinase inhibitors, as shown by our results. The 518 identified protein kinases in the human genome account for 20–30% of the drug discovery programs of many companies [36] and it is possible to characterize the target specificity of the inhibitors using panels of kinases [37].

### Limitations

One limitation of this analysis is that the bipartite model is only a first approximation of reality, since many nodes in the target layer are controllers themselves, interactions downstream of the targets can feed back to the control layer, and nodes often do interact with other nodes of the same class. Additionally, links in our model are unweighted, whereas biological interactions can be inhibitory or excitatory, with varying strength of action. It is not possible to determine theoretically which is the appropriate level of simplification for this model, which we apply both to naturally occurring biological control and to pharmacological control. Only the efficacy of the experimental interventions mentioned above will allow us to determine if any usefulness is retained. It should also be noted that these interaction datasets are incomplete, have varying levels of confidence, and are not fully validated. The quantitative patterns we have described are, however, common to datasets of very different origin and therefore cannot reasonably be explained by experimental noise or bias present in each dataset.

### Conclusion

We have shown the generality of several network metrics of biological combinatorial control. This discovery, together with our increasing understanding of the mathematical principles underlying biological control structures and their property of efficient robustness, serve as building blocks for a new approach to pharmacological control of biological systems. This approach utilizes naturally occurring drug promiscuity to build sets with biomimetic properties, such as many-to-many targeting, very wide coverage of the target set, and redundancy of incoming links per target. Importantly, these are quantitative properties of the network and cannot be described by listing features of individual drugs, such as selectivity. We therefore do not simply suggest the use of nonselective therapeutic agents but propose testing the use of drugs to build layers of control similar to those present within cells. This suggestion is also consistent with a recent paper from the Barabasi group showing that biological networks can be fully controlled only by acting on at least 80% of the nodes [38], [39]. This systems-level approach to pharmacological intervention would mimic combinatorial strategies that are ubiquitous in Nature.

## Materials and Methods

### Data and software

Predicted human microRNA-mRNA binding sites were downloaded from the TargetScan database [40] release 5.1 (http://www.targetscan.org). Only conserved targets of conserved miRNA families were used (made available in the file “Predicted_Targets_Info.txt”). Human transcription factor binding sites were gathered from the TRANSFAC database [41]. The network was trimmed for binding sites that could be mapped directly to a transcription factor with an Entrez Gene identifier (reducing 615 DNA binding domains to 389 known transcription factors and 13362 DNA binding sites to 9284 binding sites). Yeast transcription factor to gene regulations were downloaded from the YeasTRACT database [42] (http://www.yeastract.com). Human phosphorylation binding sites were downloaded from the PhosphoPOINT database [43] (http://kinase.bioinformatics.tw), using only sites in Category 3 (Known Substrate) and Category 4 (Interacting Phospho-protein with Known Substrate) [43]. Yeast phosphorylation binding sites were extracted from the Phosphorylome database [44] website (http://networks.gersteinlab.org/phosphorylome/). *E. coli* transcription factor binding sites were downloaded from the RegulonDB database [45] release 6.4 (http://regulondb.ccg.unam.mx). Parsing and formatting of the data was performed in Python, when necessary. All data analysis was performed in R. The Bioconductor suite in R was used to perform all gene annotations (“org.Hs.eg.db” package), and Gene Ontology enrichment analysis (“GOstats package”).

Numerical simulations of the mathematical model were performed in Matlab. All R and Matlab code is made available at http://paternostrolab.org/.

### Degree distribution analysis

The discrete analog to the continuous exponential distribution is the geometric distribution
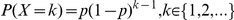
which has expected value
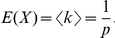
Therefore, for a distribution with known expected value 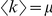, 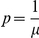.

Unlike histogram approaches, the cumulative distribution function (cdf) avoids binning effects and displays every data point. In Figure 2A, empirical cumulative distribution functions for each network had their x-axis normalized by 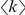 and were plotted next to the cdf of the geometric distribution
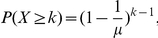
with the x range normalized by μ. Similar curves were produced by different μ>1, converging to the curve in Figure 2A for μ>>1.

### Bipartite random graph model

Figures 2B and 2C show binned histograms of the degree distribution data, compared with histograms of the null distribution expected from a bipartite modification of the Erdös-Rényi random graph model [18]. In graph theory [46], this model links any two nodes according to a probability *p*. Similarly, we can consider bipartite random networks of controllers and targets with the same number of control nodes *M* and target nodes *N* as each biological network, and with the probability *p* of a link between any control and any target node equal to the measured link density *D*. Random bipartite graphs have incoming and outgoing links according to the binomial distribution, using *D* as the probability parameter. Since the networks are large, the Poisson distribution
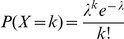
was used as an approximation to the binomial, with *λ* = *<k>*, with *<k>* = *MD* for targets and *<k>* = *ND* for controllers. The dashed curves in Figure 2B and 2C are histograms of the expected Poisson distribution of links for the M, N, and D of each network, using the same binning as the biological data.

### Sampling algorithms

In addition to the approach described in the results section, we also developed an alternative algorithm for sampling biomimetic controller sets from a large bipartite network (e.g., selecting a subset of kinase inhibitors from a pharmaceutical compound library). The algorithm selects an arbitrarily sized subset of controllers, given the desired monotonically decreasing distribution of incoming links for the target nodes and an ordered list of target nodes.

First, the target list can optionally be ordered by one or many biological criteria. In the case of the kinase inhibitor network, kinase targets can be ranked using information such as disease relevance, mutation status, protein expression, or phosphorylation state.

Next, the desired continuous link distribution p(k) is discretized to P[k] for k = [1,2,…,N], which assigns a desired integer number of incoming links for each target node. In the case of the kinase inhibitor network, this step assigns the highest incoming links P[k = 1] to the top-ranked kinase target, the second highest incoming links P[k = 2] to the second kinase in the list, and so on. In this way, the algorithm generates an incoming link profile that ensures that more important targets receive more incoming links and therefore are more likely to be inhibited or regulated.

Finally, a linear programming algorithm selects the minimal set of controllers (inhibitors) that satisfies or exceeds the incoming link profile for the set of targets (kinase). The linear programming problem is to minimize a binary vector x so that Ax≥b, where x is of the same length as the controller library and denotes whether a node is selected as part of the subset, A is the adjacency matrix describing the controller-target network links and b is the incoming link profile for each target. Since each row of A represents the connectivity of a single target node, the column vector b = Ax is the sum of incoming links from the subset x for every target in the network. Solutions to linear programming problems may be degenerate, so multiple subset solutions may be possible.

### Mathematical model of a bipartite information processing network

We neglect the feedback from targets to controllers. At the molecular level, the details of biological interactions and signal propagation are complex and idiosyncratic; therefore we used an abstract model of signaling similar to Boolean networks. In this model, control signals are represented by control node values of either 1 or 0. Links are not weighted, passing input values to the output node unaltered. Control signals reaching a target are then computed by one of three rules, and the target's output is a binary value indicating its active/inactive state. The “OR” rule designates that an output node is active if any of its connected input nodes is active. The “AND” rule requires all inputs to be active in order to activate the output node. The “MAJORITY” rule counts the number of incoming links, and activates the output node if more than half of the inputs are active, otherwise the output remains inactive. Bipartite networks using one of the three rules are studied separately. Examples can be found in the biological literature supporting the applicability of all three rules. Standard descriptions of gene control by transcription factors state that “each eukaryotic gene is therefore regulated by a committee of proteins, all of which must be present to express the gene at its proper level” [1]. In the same standard reference the analogy with a microprocessor AND gate is explicitly made [1] for intracellular signal transduction. Recent studies on multisite phosphorylation by protein kinases describe cases where a proportion of sites above a threshold number needs to be phosphorylated to switch on degradation of a protein [47], [48], a clear example of the MAJORITY rule. In the cases of miRNAs many studies have been reported describing clear effects of adding or silencing one miRNA [49], [50], which would be consistent with the OR rule. It s clear, however, that these rules are only a very simplified representation of actual biological control effects.

For a given number of controllers *M* and targets *N*, 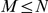, links are randomly added between controllers and targets with a probability *D*, defined as the network density, or the total links divided by the number of possible links M*N. Density can also be calculated from the relationship
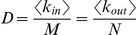
, where <*k_in_*> and <*k_out_*> are the average incoming links *k_in_* to the *N* targets and average outgoing links *k_out_* from the *M* controllers, respectively.

### Robustness

The robustness to link deletion is defined as follows: given a random bipartite network defined above, and a random binary input sequence to the controller nodes, what is the fraction of output nodes that change in response to the deletion of *γ* links? This is equivalent to asking, what is the probability that a single output node changes in response to the deleted link?

Consider a single node having a fixed number of incoming links *k_in_* and an output according to the OR rule. Define 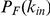 as the probability that a target node is in a “fragile” condition, meaning that deletion of one specific incoming link for that node will change the output. Deleting a link to an inactive control node will not change the output, so the only fragile state in the OR case is to have 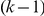 inactive, or “0”, inputs, and a single active, or “1” input, out of all 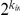 possible binary sequences of inputs. Therefore,
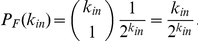



Then, the probability *F_γ_* that an output node with 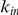 incoming links changes in response to *γ* randomly deleted links in a network containing 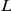 links is
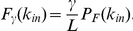



This expression takes into account that *γ/L* is the probability of hitting the “fragile” link. The robustness of a target with 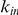 incoming links can then be defined as
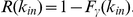



This quantity can be averaged over the target nodes by taking an expectation value over the degree distribution according to
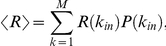
where 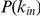 is the degree distribution of incoming links. This is the quantity plotted in Fig. 3.

### Number of output states

We define output states 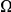 as the total number of unique binary output sequences that our bipartite network can achieve. This quantity has a maximum of 2*^M^* for a one-to-one network (see Figure 1). We can estimate 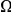 for large networks by considering first the output entropy for a single output node with *k_in_* incoming links. The single node entropy is
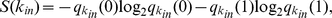
where *q_k_* is the probability of occurrence of each output state. For the “OR” rule, only when all inputs are zero is the output also inactive, therefore
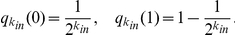
Inserting values for *q_k_*,
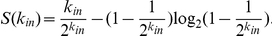
Using
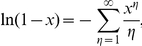
We obtain
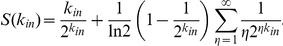



As for the robustness, we can take an expectation value of the entropy over the degree probability distribution
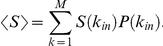
The total number of states can then be estimated using 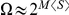. In Figure 3 we use a truncation of the series for *S*(*k_in_*) to η = 3.

### Expected values

We provide here some expressions that are useful to calculate the expectation values of the entropy and robustness over the *k_in_* degree distributions. These expectation values are calculated according to the general expression
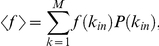
where *P*(*k_in_*) is the degree distribution of incoming links and *f* is a function of 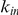.

Note that both robustness and entropy can be expressed in terms of the quantities 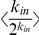 and 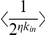, with 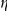 integer. These expected values can be explicitly calculated for an exponential (geometric) distribution 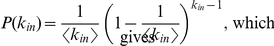


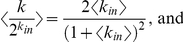


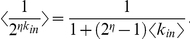



If the links are randomly distributed with 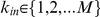, as in the bipartite random network model described above, then *P*(*k_in_*) is the binomial distribution. Assuming a large network, however, *P*(*k_in_*) is approximated by the Poisson distribution
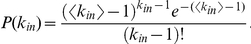



Note that we are not including 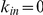 in our analysis. Using this distribution we obtain
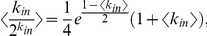
and
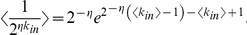



The resulting curves are similar for Poisson and exponential link distributions (see Figure S7, leading to similar optimal values 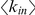 that maximize both robustness and entropy.

## Supporting Information

Text S1 **Further analysis of many-to-many control, enrichment of gene categories in target genes, overlap measures, link distributions, deviations from random networks, and maximum entropy distribution.**
(DOCX)

Figure S1 **Venn diagram of human gene targets, by types of controller molecule.** Selected top GO annotations (p-value<0.001) for each slice of the Venn diagram are listed.
(DOCX)

Figure S2 **Illustration of the two overlap terms** used in Table 1: *Pairwise overlap of targets. In this example, pairwise overlap for x_1_ with respect to x_2_ = 2/5 (40%), **Shared targets per controller. In this example, the percent of shared targets for x_3_ = 3/5 (60%).
(DOCX)

Figure S3 **Fitting controllers per target (incoming links) to an exponential distribution.** The E. coli and yeast transcription factor networks fit tightly with this distribution, while all human networks and the yeast phosphorylation network seem to have a fat-tail or scale-free component.
(DOCX)

Figure S4 **Fitting targets per controller (outgoing links) to an exponential distribution.** All but the *E. coli* transcription factor network have at least some exponential component.
(DOCX)

Figure S5 **Fitting controllers per target (incoming links) to a scale-free distribution.** The human and yeast phosphorylation networks fit more tightly with this distribution.
(DOCX)

Figure S6 **Fitting targets per controller (outgoing links) to a scale-free distribution.** The *E. coli* transcription factor network is better modeled by a scale-free distribution, and the human kinase network may also have a scale-free component.
(DOCX)

Figure S7 **Comparison of the analytical model of** **Figure 3** **for the two different link distributions.**
(DOCX)

Figure S8
**Deviations of K_C_-K_T_ correlations in each network from degree preserving random networks.** Deviations of the plotted quantity from zero indicates that a probability of finding a link connecting nodes with connectivity 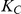 and 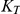 is different than for the null model. Z-score represents the difference (in standard deviations) between the biological value and the mean of 30 degree-preserving randomizations.(DOCX)

Figure S9 **Distributions of incoming and outgoing links for the simulated kinase inhibitor library and the sampled biomimetic kinase inhibitor network.**
(DOCX)

Table S1 **Presence of controller nodes of each type in the target sets of human networks.** Transcription factors and kinases were significantly enriched in the target sets of all three networks. For example, of the 389 transcription factors from the human TF network, 147 were found in the target set of the miRNA network.
(DOCX)

Table S2 **Top 10 over-represented GO Biological Process terms for highly targeted genes** in three human networks (genes with incoming links greater than 5 times the network average). Bold denotes appearance in more than one network (even if not shown in the top 10). Size is the number of target genes in both subsets that are associated with the GO term. ExpCount is expected number of appearances of the term and Count is the actual number of appearances.
(DOCX)

Table S3 **Top 10 over-represented GO Biological Process terms for low-degree genes** in three human networks (genes with incoming links less than twice the network average). Bold denotes appearance in more than one network. Size is the number of target genes in both subsets that are associated with the GO term.
(DOCX)

Table S4 **Comparison of overlap parameters in biological networks to random.** Shuffled networks have equal numbers of nodes and links, as well as equivalent link distributions, as the biological network. Random networks have equal numbers of nodes and links, with links placed randomly. Shared Targets per Controller (STC) and Pairwise Overlap (PO) measurements are presented as mean over all controllers or pairs of controllers, respectively. Mean and standard deviation of overlap parameters in the shuffled and random networks varied less than 2% over 5 simulations.
(DOCX)

Table S5 **Evaluation of fitting models.** Higher R-squared values for each network are in bold.
(DOCX)
