# Supplementary material for: Statistical Properties and Robustness of Biological Controller-Target Networks
Source: PLoS One. 2012 Jan 3;7(1):e29374. doi: 10.1371/journal.pone.0029374 (PMC3250441; doi:10.1371/journal.pone.0029374)
Supplement: Text S1 — Further analysis of many-to-many control, enrichment of gene categories in target genes, overlap measures, link distributions, deviations from random networks, and maximum entropy distribution. (DOCX) [file pone.0029374.s001.docx]

**Supporting Text S1**

[S1.1 Additional examples of many-to-many control in biology 1](#_Toc311146788)

[S1.2. Enrichment of gene categories in network targets 2](#_Toc311146789)

[S1.2.1 Enrichment of controller nodes in target sets by network 2](#_Toc311146790)

[S1.2.2 Unbiased GO enrichment of target nodes separated by k_in_ 2](#_Toc311146791)

[S1.2.3 Enrichment of target nodes by number of controller types 3](#_Toc311146792)

[S1.3. Analysis of overlap measures 3](#_Toc311146793)

[S1.3.1 Overlap measures in biological networks and in random bipartite networks 3](#_Toc311146794)

[S1.3.2 Derivation of overlap measures in random networks 4](#_Toc311146795)

[S1.4. Further analysis of link distributions 6](#_Toc311146796)

[S1.4.1 Fitting incoming/outgoing links to exponential and scale-free distributions 6](#_Toc311146797)

[S1.4.2. Exponential distributions cannot be obtained by sampling a scale-free network 6](#_Toc311146798)

[S1.4.3 Effect of link distribution on the analytical network model 6](#_Toc311146799)

[S1.5. Deviations from random degree-preserving networks 7](#_Toc311146800)

[S1.6. Maximum entropy distribution 7](#_Toc311146801)

[S1.7. Supplementary References 8](#_Toc311146802)

## S1.1. Additional examples of many-to-many control in biology

A many-to-many combinatorial structure is not limited to the control of cells and it is found in all types of complex control in biology, the most striking example being the control of the organism by the nervous system, where connections among neurons have a many-to-many arrangement. The control of effectors by neurons has a simpler structure, as shown by motor units [[6](#_ENREF_6)], where each motor neuron controls a distinct set of muscle fibers and the target sets are not overlapping, in a one-to-many fashion. The complexity of control structure might depend on the complexity of the target system.

Diseases such as cancer may also adapt by developing combinatorial strategies to counter intrinsic defense mechanisms and homeostatic reactions or extrinsic therapeutic interventions [[7](#_ENREF_7)]. An increasing body of evidence shows that the resistance of cancer to therapies involves molecules acting at multiple levels with many-to-many actions. This provides further support for the use of biomimetic therapeutic strategies of matching complexity.

## S1.2 Enrichment of gene categories in network targets

Since target nodes have a broad distribution of incoming control links, we used the three human networks to explore whether certain categories of nodes may be more highly targeted than others. Controller nodes appeared in the target sets more than expected by random (Section S1.2.1). Highly targeted genes in all networks shared many significantly enriched Gene Ontology [[4](#_ENREF_4)] (GO) terms involved in transcription, regulation, and development (Section S1.2.2). Conversely, sparsely targeted genes tended to be enriched in GO terms involving biological “effector” processes, such as metabolism, transport, and the response to stimulus. Additionally, human genes regulated by all three types of controller molecule were almost always themselves involved in regulation (Section S1.2.3).

### S1.2.1 Enrichment of controller nodes in target sets by network

We examined the enrichment of controller nodes from each human network within all target sets. Kinases and transcription factors can be targets for regulation by all three controller networks, whereas microRNAs are not directly regulated by other microRNAs and cannot be phosphorylated by kinases (NA in Table S1). MicroRNAs are known to be regulated by transcription factors, but to our knowledge large-scale binding data are not available. Transcription factors in TRANSFAC were mapped to Entrez gene numbers where possible by both automatic and manual methods, resulting in a trimmed list of 197 transcription factors. All kinases were mapped to Entrez gene identifiers in the original PhosphoPOINT database. Table S1 shows the representation of kinases and transcription factors as targets in the three networks.

Enrichment of transcription factors and kinases (control nodes from their respective network databases) in target sets of each of the networks were found to be significant by hypergeometric tests in a gene universe of 20,500, resulting in p-values below the computational lower limit in each case. The analysis was repeated against the full set of transcription factors and kinases in the human genome, as identified by GO annotation (both GO:0003677 – “DNA binding” and GO:0003700 – “transcription factor activity” for transcription factors; and GO: 0004672 – “protein kinase activity” for kinases), and these gene sets were similarly found to be significantly enriched in all target sets in all cases.

### S1.2.2 Unbiased GO enrichment of target nodes separated by k_in_

For each network, we divided target nodes into two subsets based on the number of linked controllers: “highly targeted” genes with greater than 5 times the mean incoming links of the network, and “sparsely targeted” genes with less than half the mean incoming links of the network. Each subset was then subjected to unbiased Gene Ontology (GO) enrichment analysis to find over-represented gene categories. The top 10 GO terms that were enriched in the highly-targeted subset are shown in Table S2. Over-represented GO terms for the sparsely-targeted subset of genes are presented in Table S3. Here, non-coding RNA metabolism appeared in the top GO terms for both the microRNA and transcription factor networks. Oxidation reduction was the only other shared term, while the phosphorylation network did not have any significantly over-represented terms in its low-degree target proteins (p-value threshold = 0.001). As discussed in the main text, GO terms involved in “regulation” were more prevalent in the highly-targeted set, while genes that can be thought of as “effectors” (e.g. metabolic genes, transporters, and response genes) were enriched in the sparsely-targeted set.

### S1.2.3 Enrichment of target nodes by number of controller types

Next, we separated genes by the number of different *types* of control molecule targeting a given gene. Figure S1 is a Venn diagram of all human genes with GO annotations, separated into groups targeted by 0, 1, 2 or all 3 of the cellular control networks. Genes not targeted by any network were enriched in GO annotations involved in neurological, immune, and G-protein signaling systems, which might be more indicative of our incomplete knowledge of these networks. Genes targeted by all three types of controller molecule were often involved in regulation themselves, much like the highly targeted genes in Table S3. Genes targeted by only one of the miRNA, transcription factor, or kinase networks have unique properties that are often related to the type of controller, for example genes targeted only by miRNAs are more likely to be involved in RNA splicing, and kinase targets are enriched in genes annotated as “intracellular signaling cascade.”

## S1.3. Analysis of overlap measures

We devised two measures to quantify the amount of overlap among target sets, reflecting the extent to which a set of target nodes is combinatorially regulated. “Shared Targets per Controller” (STC) is defined as the average percentage of a controller’s targets that have more than one incoming link, and “Pairwise Overlap” (PO) is defined as the average percent of targets shared between any given pair of controllers (see Figure S2).

### S3.1 Overlap measures in biological networks and in random bipartite networks

All networks had high “Shared Targets per Controller” (STC), meaning that any control node shares the vast majority of its targets with at least one other controller. The mean “Pairwise Overlap” PO between any two controllers was an average of 5.7% ± 2.6% over all networks (Table 1 in the main text). Using the random bipartite graph framework, we derived the following equations for the expected values of STC and PO in bipartite random networks as a function of *M*, *N*, and *D* (derivations in the next section):

*E*[*STC*] *=* [1 – (1 – *D*)*^N^*]*[1 – (1 – *D*)*^M –^* ^1^] ,$E[{STC]=[1-(1-D)}^{N}]*[1-\left( 1-D \right)^{M-1}]$

*E*[*PO*] = [1 – (1 – *D*)*^N^*]**D .*

$E[PO]=\left[ 1-\left( 1-D \right)^{N} \right]*D$

For each biological network, we then compared the calculated overlap parameters to the values expected for random bipartite graphs of equal size. We also generated shuffled versions of the networks by swapping links while retaining the original link-per-node distributions (Table S4). We found that average values for Shared Targets per Controller were significantly lower in biological systems than in their random counterparts. Pairwise Overlap was significantly higher in the biological networks except for the *E. coli* transcription factors. Both metrics were mostly explained by the link distributions, as evidenced by the similarities between the biological and the shuffled networks.

### S1.3.2 Derivation of overlap measures in random networks

Let us consider an ensemble of bipartite directed network characterized by $M$ controllers and $N$ targets. Many properties of the ensemble can be defined in terms of the distribution for the number of links out of a controller node, *p*(*N*,*k_out_*), and the distribution for the number of links into a target node, *q*(*M*,*k_in_*). We will focus on the Erdös-Rényi random graph model [[1](#_ENREF_1)], for which these two distributions read

$p\left(N,k_{out}\right)=\left(\begin{matrix} N \\ k_{out} \end{matrix}\right)D^{k_{out}}\left(1-D\right)^{N-k_{out}}$,

$q\left(M,k_{in}\right)=\left(\begin{matrix} M \\ k_{in} \end{matrix}\right)D^{k_{in}}\left(1-D\right)^{M-k_{in}}$,

where$D$ is the probability to have a link.

The Shared Targets per Controller (STC) is a quantity that characterizes how likely a target reached by a controller is shared with other controllers. This can be calculated by fixing one particular controller out of the $M$ available and defining the quantity

$z=\sum_{k_{in}=1}^{M-1} q\left(M-1,k_{in}\right)$.

The quantity *z* is the total probability that at least one of the other *M –* 1 controllers is co-controlling a target reached by the initially fixed controller. If the initial controller has *k_out_* links out, then the Shared Targets per Controllers is

$STC\left(k_{out}\right)=\frac{1}{k_{out}}\sum_{m=1}^{k_{out}} m\left(\begin{matrix} k_{out} \\ m \end{matrix}\right)z^{m}\left(1-z\right)^{k_{out}-m}$,

where $1/k_{out}$ is a normalization factor, and the binomial coefficient takes into account in how many ways $k_{out}$targets can be co-controlled by additional $m$controllers. The final expression for the STC can be written as

$STC=\sum_{k_{out}}^{N} p\left(N,k_{out}\right)STC\left(k_{out}\right)$,

which in the random model leads to the simple expression

$STC=\left[1-\left(1-D\right)^{N}\right]\left[1-\left(1-D\right)^{M-1}\right]$,

since $STC\left(k_{out}\right)=z$ does not depend on *k_out_* in this case.

The Pairwise Overlap (PO) is a quantity that characterizes the probability that two controllers overlap by acting on the same target. This quantity can be calculated in a similar way as the STC by fixing a pair of controller nodes. Then we can define the quantity

$h=\sum_{k_{in}=1}^{M-1} q\left(M-1,k_{in}\right)\frac{k_{in}}{M-1}$,

which gives the total probability that one node at the end of the link from the first controller is also connected to the second controller in the pair. If the initial controller has *k_out_* links out, then the Pairwise Overlap can be explicitly expressed in terms of *h* as

$PO\left(k_{out}\right)=\frac{1}{k_{out}}\sum_{m=1}^{k_{out}} m\left(\begin{matrix} k_{out} \\ m \end{matrix}\right)h^{m}\left(1-h\right)^{k_{out}-m}$,

and the total PO is

$PO=\sum_{k_{out}=1}^{N} p\left(N,k_{out}\right)PO\left(k_{out}\right)$.

In the random graph case we have$PO\left(k_{out}\right)=h=D$, which leads to the final result

$PO=\left[1-\left(1-D\right)^{N}\right]D$.

## S1.4. Further analysis of link distributions

### S1.4.1 Fitting incoming/outgoing links to exponential and scale-free distributions

Cumulative distributions of links per node were compared to each other in Figure 2A of the main text, while histograms of incoming and outgoing links in the biological control networks were compared against exponential and binomial distributions in Figure 2B and 2C. Here we fit the empirical cumulative distribution function for each individual dataset against both exponential and scale-free distributions, and measure the goodness of fit.

Figures S3 and S4 fit the curves of Figure 2 to an exponential distribution by plotting$y(x)=-\ln\left(1-F(x)\right)$, where *F(x)* is the empirical cumulative distribution function (cdf). For the exponential distribution, $F\left(x\right)=\Pr(X>x)=1-e^{-\lambda x}$ and $y(x)=-\ln\left(1-F\left(x\right)\right)=\lambda x$; therefore, the goodness of fit is given by the R^2^ of a least-squares linear fit to *y(x)*.

Figures S5 and S6 fit the curves of Figure 2 to a power-law distribution. In this case, $F\left(x\right)=\Pr(X>x)=1-x^{-\gamma+1}$, and we similarly take the log transform $y(x)=-\ln\left(1-F(x)\right)$, resulting in $y(x)=-\ln\left(1-F(x)\right)=-\ln(x^{-\gamma+1})=\left(\gamma-1\right)\ln(x)$. Therefore, an additional logarithmic transform of the x-axis yields a straight line if the data are power-law distributed.

The microRNA and all three transcription factor networks seem to better fit an exponential distribution, while the phosphorylation networks in both human and yeast may have some scale-free component. The yeast and E. coli transcription factor networks are well-modeled by exponential distributions. Table S5 compares all fits using R^2^ values. For both types of links, the human kinase network is a better fit to a scale-free distribution. With the exception of the human transcription factor network, all networks and link types fit at least one distribution with an R^2^ of > 0.9.

### S1.4.2. Exponential distributions cannot be obtained by sampling a scale-free network

To detect the possibility that the exponential distribution was an artifact of sampling from a scale-free network, we generated a scale-free network in-silico using the Barabasi-Albert (B-A) growth and preferential attachment model [[5](#_ENREF_5)], and then randomly assigned nodes to control and target layers, keeping all links between the two layers. A bipartite network was also generated de-novo by constraining the B-A model to operate with two node classes and unidirectional links. In both cases, the resulting bipartite networks followed a scale-free distribution rather than an exponential.

### S1.4.3 Effect of link distribution on the analytical network model

As shown in the methods, analytical expressions for robustness and output states (as a function of the entropy of the output node) can be computed for different distributions of links. Since the biological networks are shown to be exponentially distributed, it is important to examine the effect of the distribution on the relationship to <*k_in_*>. In Figure S7, we show that the curves are similar for the exponential and Poisson distributions, and do not alter the main conclusions of the paper.

## S1.5. Deviations from random degree-preserving networks

In order to detect correlations in the connectivity of the nodes, we have calculated the probability $P\left(K_{C},K_{T}\right)$ that a link connects a controller node with degree $K_{C}$ to a target node with degree$K_{T}$. This quantity has been compared to a null-model obtained by randomizing the network using an algorithm that preserves degree distributions [[2](#_ENREF_2)] [[3](#_ENREF_3)]. In Figure S8, we show this comparison for the seven control networks examined in this study. We plot in Figure S8 the statistical significance of $P\left(K_{C},K_{T}\right)$, for binned values of $K_{C}$ and $K_{T}$, in comparison with a null-model randomized case $P_{R}\left(K_{C},K_{T}\right)$ , averaged over 30 degree-preserving randomized configurations. In the plot, $Z\left(K_{C},K_{T}\right)=\frac{\left(P\left(K_{C},K_{T}\right)-\overline{P_{R}\left(K_{C},K_{T}\right)}\right)}{\sigma\left(K_{C},K_{T}\right)}$, where $\sigma\left(K_{C},K_{T}\right)$ is the standard deviation of $P_{R}\left(K_{C},K_{T}\right)$ in the 30 random realizations. Any deviation of this ratio from zero indicates the presence of correlations in the connectivity of the biological network that would not be present in random networks with the same degree distribution of the real network.

Note that biological networks seem to have more correlation structure than the chemically-based kinase inhibitor network. For example, biological networks have some preference for links further out along the axes. The physical interpretation of this pattern is that controllers with many output links are preferentially connected to targets with few input links, and vice-versa (compared to random networks with equivalent degree distributions). A similar pattern of correlations was also observed in an analysis of the transcription regulatory network of yeast [[2](#_ENREF_2)]. Although these in-degree/out-degree correlation patterns are not as robustly conserved as the other statistical properties that we focus on in the main text, the analysis reveals trends that may be interesting avenues for future research.

## S1.6. Maximum entropy distribution

The “maximum entropy distribution,” for a random variable with specified mean and unspecified variance, is an exponential distribution [[8](#_ENREF_8)]. For example, the height of a molecule in a gas under gravitational force has its mean constrained but no limit to its variance, and thus is exponentially distributed under thermodynamic equilibrium. Therefore, any evolving system that conserves the mean but not the variance of some observable sequence of variables (i.e., links per node), will maximize entropy of that sequence over time, regardless of the underlying processes, and eventually reach an exponential distribution [[9](#_ENREF_9)]. This is simply because these distributions are vastly more common in the space of all possible sequences with specified mean and unspecified variance. While there may be many alternative explanations for the observed distributions, one possibility is that the exponential distribution in links per node is due to maximum entropy, in other words the average links per node is tightly conserved (specified mean), while the number of links in any single node is unlimited (unspecified variance).

Other genome-wide interaction networks, such as protein-protein interaction, metabolic, and gene co-expression networks, also have distributions of links that deviate from the random graph case [[10](#_ENREF_10),[11](#_ENREF_11),[12](#_ENREF_12),[13](#_ENREF_13)], but these are generally scale-free rather than exponential distributions. Scale-free distributions can also arise by maximum entropy with unconstrained variance, but these require conservation of the geometric, not the arithmetic, mean [[14](#_ENREF_14)]. We ensured that exponential distributions cannot be obtained simply by sampling a larger, scale-free network (see section S1.3.2).

## S1.7. Supplementary References

1. Newman MEJ, Strogatz SH, Watts DJ (2001) Random graphs with arbitrary degree distributions and their applications. Physical Review E 64.

2. Maslov S, Sneppen K (2002) Specificity and stability in topology of protein networks. Science 296: 910-913.

3. Shen-Orr SS, Milo R, Mangan S, Alon U (2002) Network motifs in the transcriptional regulation network of Escherichia coli. Nat Genet 31: 64-68.

4. Ashburner M, Ball CA, Blake JA, Botstein D, Butler H, et al. (2000) Gene ontology: tool for the unification of biology. The Gene Ontology Consortium. Nat Genet 25: 25-29.

5. Barabasi, Albert (1999) Emergence of scaling in random networks. Science (New York, NY) 286: 509-512.

6. Monti RJ, Roy RR, Edgerton VR (2001) Role of motor unit structure in defining function. Muscle & Nerve 24: 848-866.

7. Zhou J (2009) Multi-Drug Resistance in Cancer: Springer-Verlag GmbH.

8. Cover TM, Thomas JA (1991) Elements of information theory. New York: Wiley. xxii, 542 p. p.

9. Frank SA (2009) The common patterns of nature. Journal of Evolutionary Biology 22: 1563-1585.

10. Jeong H, Tombor B, Albert R, Oltvai ZN, Barabasi AL (2000) The large-scale organization of metabolic networks. Nature 407: 651-654.

11. Ravasz E, Somera AL, Mongru DA, Oltvai ZN, Barabasi AL (2002) Hierarchical organization of modularity in metabolic networks. Science 297: 1551-1555.

12. Stuart JM, Segal E, Koller D, Kim SK (2003) A gene-coexpression network for global discovery of conserved genetic modules. Science 302: 249-255.

13. Yook SH, Oltvai ZN, Barabasi AL (2004) Functional and topological characterization of protein interaction networks. Proteomics 4: 928-942.

14. Bhardwaj N, Carson MB, Abyzov A, Yan K-K, Lu H, et al. (2010) Analysis of combinatorial regulation: scaling of partnerships between regulators with the number of governed targets. PLoS Computational Biology 6: e1000755-e1000755.
