# Supplementary material for: Statistical Properties and Robustness of Biological Controller-Target Networks
Source: PLoS One. 2012 Jan 3;7(1):e29374. doi: 10.1371/journal.pone.0029374 (PMC3250441; doi:10.1371/journal.pone.0029374)
Supplement: Figure S1 — Venn diagram of human gene targets, by types of controller molecule. Selected top GO annotations (p-value<0.001) for each slice of the Venn diagram are listed. (DOCX) [file pone.0029374.s002.docx]

**Figure S1: Venn diagram of human gene targets, by types of controller molecule.** Selected top GO annotations (p-value < 0.001) for each slice of the Venn diagram are listed.
