# Supplementary material for: Statistical Properties and Robustness of Biological Controller-Target Networks
Source: PLoS One. 2012 Jan 3;7(1):e29374. doi: 10.1371/journal.pone.0029374 (PMC3250441; doi:10.1371/journal.pone.0029374)
Supplement: Figure S2 — Illustration of the two overlap terms used in Table 1: *Pairwise overlap of targets. In this example, pairwise overlap for x1 with respect to x2 = 2/5 (40%), **Shared targets per controller. In this example, the percent of shared targets for x3 = 3/5 (60%). (DOCX) [file pone.0029374.s003.docx]

**Figure S2: Illustration of the two overlap terms** used in Table 1: **Pairwise overlap* of targets. In this example, pairwise overlap for x_1_ with respect to x_2_ = 2/5 (40%), ***Shared targets per controller*. In this example, the percent of shared targets for x_3_ = 3/5 (60%)
