# Supplementary material for: Statistical Properties and Robustness of Biological Controller-Target Networks
Source: PLoS One. 2012 Jan 3;7(1):e29374. doi: 10.1371/journal.pone.0029374 (PMC3250441; doi:10.1371/journal.pone.0029374)
Supplement: Figure S3 — Fitting controllers per target (incoming links) to an exponential distribution. The E. coli and yeast transcription factor networks fit tightly with this distribution, while all human networks and the yeast phosphorylation network seem to have a fat-tail or scale-free component. (DOCX) [file pone.0029374.s004.docx]

Figure S3: Fitting controllers per target (incoming links) to an exponential distribution. The E. coli and yeast transcription factor networks fit tightly with this distribution, while all human networks and the yeast phosphorylation network seem to have a fat-tail or scale-free component.
