# Supplementary material for: Statistical Properties and Robustness of Biological Controller-Target Networks
Source: PLoS One. 2012 Jan 3;7(1):e29374. doi: 10.1371/journal.pone.0029374 (PMC3250441; doi:10.1371/journal.pone.0029374)
Supplement: Figure S4 — Fitting targets per controller (outgoing links) to an exponential distribution. All but the E. coli transcription factor network have at least some exponential component. (DOCX) [file pone.0029374.s005.docx]

Figure S4: Fitting targets per controller (outgoing links) to an exponential distribution**.** All but the *E. coli* transcription factor network have at least some exponential component.
