# Supplementary material for: Statistical Properties and Robustness of Biological Controller-Target Networks
Source: PLoS One. 2012 Jan 3;7(1):e29374. doi: 10.1371/journal.pone.0029374 (PMC3250441; doi:10.1371/journal.pone.0029374)
Supplement: Figure S5 — Fitting controllers per target (incoming links) to a scale-free distribution. The human and yeast phosphorylation networks fit more tightly with this distribution. (DOCX) [file pone.0029374.s006.docx]

Figure S5: Fitting controllers per target (incoming links) to a scale-free distribution**.** The human and yeast phosphorylation networks fit more tightly with this distribution.
