# Supplementary material for: Statistical Properties and Robustness of Biological Controller-Target Networks
Source: PLoS One. 2012 Jan 3;7(1):e29374. doi: 10.1371/journal.pone.0029374 (PMC3250441; doi:10.1371/journal.pone.0029374)
Supplement: Figure S6 — Fitting targets per controller (outgoing links) to a scale-free distribution. The E. coli transcription factor network is better modeled by a scale-free distribution, and the human kinase network may also have a scale-free component. (DOCX) [file pone.0029374.s007.docx]

Figure S6: Fitting targets per controller (outgoing links) to a scale-free distribution**.** The *E. coli* transcription factor network is better modeled by a scale-free distribution, and the human kinase network may also have a scale-free component.

**
