# Supplementary material for: Statistical Properties and Robustness of Biological Controller-Target Networks
Source: PLoS One. 2012 Jan 3;7(1):e29374. doi: 10.1371/journal.pone.0029374 (PMC3250441; doi:10.1371/journal.pone.0029374)
Supplement: Figure S7 — Comparison of the analytical model of Figure 3 for the two different link distributions. (DOCX) [file pone.0029374.s008.docx]

**Figure S7: Comparison of the analytical model of Figure 3 for the two different link distributions. In this figure is the mutation rate.**
