# Supplementary material for: Statistical Properties and Robustness of Biological Controller-Target Networks
Source: PLoS One. 2012 Jan 3;7(1):e29374. doi: 10.1371/journal.pone.0029374 (PMC3250441; doi:10.1371/journal.pone.0029374)
Supplement: Figure S8 — Deviations of KC-KT correlations in each network from degree preserving random networks. Deviations of the plotted quantity from zero indicates that a probability of finding a link connecting nodes with connectivity and is different than for the null model. Z-score represents the difference (in standard deviations) between the biological value and the mean of 30 degree-preserving randomizations. (DOCX) [file pone.0029374.s009.docx]

**Figure S8.** Deviations of K_C_-K_T_ correlations in each network from degree preserving random networks. Deviations of the plotted quantity from zero indicates that a probability of finding a link connecting nodes with connectivity $K_{C}$ and $K_{T}$ is different than for the null model. Z-score represents the difference (in standard deviations) between the biological value and the mean of 30 degree-preserving randomizations.
