# Supplementary material for: Statistical Properties and Robustness of Biological Controller-Target Networks
Source: PLoS One. 2012 Jan 3;7(1):e29374. doi: 10.1371/journal.pone.0029374 (PMC3250441; doi:10.1371/journal.pone.0029374)
Supplement: Figure S9 — Distributions of incoming and outgoing links for the simulated kinase inhibitor library and the sampled biomimetic kinase inhibitor network. (DOCX) [file pone.0029374.s010.docx]

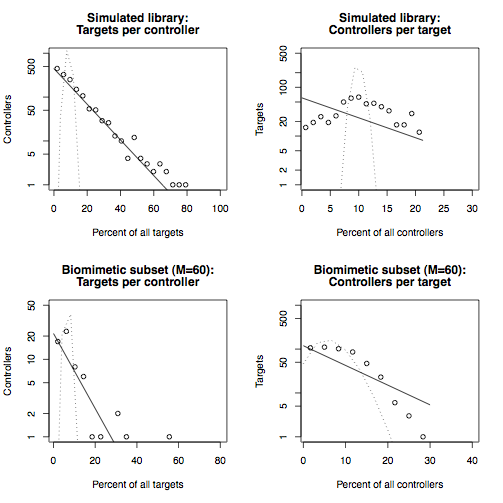


**Figure S9:** Distributions of incoming and outgoing links for the simulated kinase inhibitor library and the sampled biomimetic kinase inhibitor network. Upper panels: distributions of outgoing links from controllers (left) and incoming links per target (right) for the simulated M=1500 kinase inhibitor library acting on N=518 kinase targets. Lower panels: distributions of outgoing links from controllers (left) and incoming links per target (right) for a sampled biomimetic subset , obtained from the simulated library using the rejection method. More details for this dataset are in Results, within the "Drug-target network" section. The graphs format is the same as in Figure 2, Panels B and C.
