# Supplementary material for: Statistical Properties and Robustness of Biological Controller-Target Networks
Source: PLoS One. 2012 Jan 3;7(1):e29374. doi: 10.1371/journal.pone.0029374 (PMC3250441; doi:10.1371/journal.pone.0029374)
Supplement: Table S1 — Presence of controller nodes of each type in the target sets of human networks. Transcription factors and kinases were significantly enriched in the target sets of all three networks. For example, of the 389 transcription factors from the human TF network, 147 were found in the target set of the miRNA network. (DOCX) [file pone.0029374.s011.docx]

Table S1: Presence of controller nodes of each type in the target sets of human networks. Transcription factors and kinases were significantly enriched in the target sets of all three networks. For example, of the 389 transcription factors from the human TF network, 147 were found in the target set of the miRNA network.

|  | **Number of controllers in target set**  **(total controller pool)** | | | |
| --- | --- | --- | --- | --- |
| **Network** | *miRNA (153)* | *TF (389)* | *Kinase (264)* | *All targets* |
| *miRNA* | NA | 147 | 206 | 9448 |
| *TF* | no data | 151 | 201 | 9284 |
| *Kinase* | NA | 55 | 167 | 988 |
