# Supplementary material for: Statistical Properties and Robustness of Biological Controller-Target Networks
Source: PLoS One. 2012 Jan 3;7(1):e29374. doi: 10.1371/journal.pone.0029374 (PMC3250441; doi:10.1371/journal.pone.0029374)
Supplement: Table S2 — Top 10 over-represented GO Biological Process terms for highly targeted genes in three human networks (genes with incoming links greater than 5 times the network average). Bold denotes appearance in more than one network (even if not shown in the top 10). Size is the number of target genes in both subsets that are associated with the GO term. ExpCount is expected number of appearances of the term and Count is the actual number of appearances. (DOCX) [file pone.0029374.s012.docx]

**Table S2: Top 10 over-represented GO Biological Process terms for highly targeted genes** in three human networks (genes with incoming links greater than 5 times the network average). Bold denotes appearance in more than one network (even if not shown in the top 10). Size is the number of target genes in both subsets that are associated with the GO term. ExpCount is expected number of appearances of the term and Count is the actual number of appearances

| ***Highly targeted: > 5*mean(controls per target)*** | | | | | |  |  |  |
| --- | --- | --- | --- | --- | --- | --- | --- | --- |
| GOBPID | Pvalue | ExpCount | Count | Size | Term |  |  |  |
| *TF* |  |  |  |  |  |  |  |  |
| GO:0080090 | 1.0E-59 | 227.5 | 441 | 1655 | **regulation of primary metabolic process** | | | |
| GO:0060255 | 3.4E-58 | 230.7 | 442 | 1678 | **regulation of macromolecule metabolic process** | | | |
| GO:0032774 | 1.7E-50 | 127.8 | 290 | 930 | **RNA biosynthetic process** | | |  |
| GO:0034961 | 8.6E-49 | 233.3 | 426 | 1697 | **cellular biopolymer biosynthetic process** | | | |
| GO:0065007 | 2.1E-47 | 524.4 | 732 | 3815 | biological regulation | | |  |
| GO:0009059 | 4.0E-43 | 249.5 | 433 | 1815 | **macromolecule biosynthetic process** | | | |
| GO:0009889 | 3.5E-42 | 113.6 | 253 | 907 | **regulation of biosynthetic process** | | | |
| GO:0010467 | 9.9E-42 | 262.4 | 445 | 1909 | **gene expression** | |  |  |
| GO:0051171 | 1.7E-41 | 110.3 | 247 | 882 | **regulation of nitrogen compound metabolic process** | | | |
| GO:0031323 | 3.3E-41 | 128.9 | 272 | 1037 | **regulation of cellular metabolic process** | | | |
|  |  |  |  |  |  |  |  |  |
| *microRNA* | |  |  |  |  |  |  |  |
| GO:0060255 | 1.9E-16 | 267.3 | 377 | 1896 | **regulation of macromolecule metabolic process** | | | |
| GO:0080090 | 3.0E-16 | 268.8 | 378 | 1907 | **regulation of primary metabolic process** | | | |
| GO:0009889 | 3.2E-15 | 239.4 | 341 | 1698 | **regulation of biosynthetic process** | | | |
| GO:0031323 | 4.9E-14 | 280.5 | 382 | 1990 | **regulation of cellular metabolic process** | | | |
| GO:0051171 | 1.4E-13 | 230.9 | 325 | 1638 | **regulation of nitrogen compound metabolic process** | | | |
| GO:0044260 | 4.6E-12 | 460.7 | 562 | 3268 | **cellular macromolecule metabolic process** | | | |
| GO:0050789 | 8.7E-12 | 534.4 | 634 | 3791 | regulation of biological process | | | |
| GO:0034961 | 2.6E-11 | 254.2 | 341 | 1803 | **cellular biopolymer biosynthetic process** | | | |
| GO:0009059 | 1.5E-08 | 213.6 | 282 | 1556 | **macromolecule biosynthetic process** | | | |
| GO:0043283 | 6.4E-08 | 328.2 | 399 | 2422 | **biopolymer metabolic process** | | |  |
|  |  |  |  |  |  |  |  |  |
| *Kinase* |  |  |  |  |  |  |  |  |
| GO:0034961 | 4.9E-04 | 3.3 | 9 | 281 | **cellular biopolymer biosynthetic process** | | | |
| GO:0009059 | 6.1E-04 | 3.3 | 9 | 288 | **macromolecule biosynthetic process** | | | |
| GO:0044419 | 8.2E-04 | 0.9 | 5 | 74 | interspecies interaction between organisms | | | |
| GO:0006355 | 9.7E-04 | 2.0 | 7 | 171 | **regulation of transcription, DNA-dependent** | | | |
