# Supplementary material for: Statistical Properties and Robustness of Biological Controller-Target Networks
Source: PLoS One. 2012 Jan 3;7(1):e29374. doi: 10.1371/journal.pone.0029374 (PMC3250441; doi:10.1371/journal.pone.0029374)
Supplement: Table S3 — Top 10 over-represented GO Biological Process terms for low-degree genes in three human networks (genes with incoming links less than twice the network average). Bold denotes appearance in more than one network. Size is the number of target genes in both subsets that are associated with the GO term. (DOCX) [file pone.0029374.s013.docx]

**Table S3: Top 10 over-represented GO Biological Process terms for low-degree genes** in three human networks (genes with incoming links less than twice the network average). Bold denotes appearance in more than one network. Size is the number of target genes in both subsets that are associated with the GO term.

| **Low targeted: 0.5*mean(controls per target)** | | | | | |  |  |
| --- | --- | --- | --- | --- | --- | --- | --- |
| GOBPID | Pvalue | ExpCount | Count | Size | Term |  |  |
| *TF* |  |  |  |  |  |  |  |
| GO:0034660 | 1.1E-05 | 40.51129 | 62 | 101 | **ncRNA metabolic process** | | |
| GO:0022900 | 1.2E-05 | 27.27493 | 45 | 68 | electron transport chain | | |
| GO:0055114 | 2.1E-05 | 91.28796 | 122 | 229 | **oxidation reduction** | |  |
| GO:0044271 | 6.4E-05 | 33.29146 | 51 | 83 | nitrogen compound biosynthetic process | | |
| GO:0042773 | 7.8E-05 | 13.63747 | 25 | 34 | ATP synthesis coupled electron transport | | |
| GO:0042180 | 8.4E-05 | 111.1052 | 142 | 277 | cellular ketone metabolic process | | |
| GO:0006120 | 9.7E-05 | 11.63196 | 22 | 29 | mitochondrial electron transport, NADH to ubiquinone | | |
| GO:0006968 | 9.7E-05 | 11.63196 | 22 | 29 | cellular defense response | | |
| GO:0006732 | 1.5E-04 | 27.67603 | 43 | 69 | coenzyme metabolic process | | |
| GO:0022613 | 1.6E-04 | 31.68705 | 48 | 79 | ribonucleoprotein complex biogenesis | | |
|  |  |  |  |  |  |  |  |
| *miRNA* |  |  |  |  |  |  |  |
| GO:0055114 | 2.5E-10 | 97.46176 | 145 | 240 | **oxidation reduction** | |  |
| GO:0034660 | 6.4E-10 | 27.20807 | 52 | 67 | **ncRNA metabolic process** | | |
| GO:0007600 | 2.0E-06 | 70.65978 | 101 | 174 | sensory perception | |  |
| GO:0006955 | 5.7E-06 | 85.33713 | 117 | 211 | immune response | |  |
| GO:0022613 | 1.3E-05 | 25.17762 | 42 | 62 | ribonucleoprotein complex biogenesis | | |
| GO:0006396 | 4.4E-05 | 109.2384 | 141 | 269 | RNA processing | |  |
| GO:0006364 | 5.8E-05 | 8.121813 | 17 | 20 | rRNA processing | |  |
| GO:0007601 | 6.3E-05 | 42.23343 | 62 | 104 | visual perception | |  |
| GO:0006091 | 1.2E-04 | 60.50751 | 83 | 149 | generation of precursor metabolites and energy | | |
| GO:0045087 | 0.0007 | 21.11671 | 33 | 52 | innate immune response | | |
|  |  |  |  |  |  |  |  |
| *Kinase* |  |  |  |  |  |  |  |
| none |  |  |  |  |  |  |  |
|  |  |  |  |  |  |  |  |
