# Supplementary material for: Statistical Properties and Robustness of Biological Controller-Target Networks
Source: PLoS One. 2012 Jan 3;7(1):e29374. doi: 10.1371/journal.pone.0029374 (PMC3250441; doi:10.1371/journal.pone.0029374)
Supplement: Table S4 — Comparison of overlap parameters in biological networks to random. Shuffled networks have equal numbers of nodes and links, as well as equivalent link distributions, as the biological network. Random networks have equal numbers of nodes and links, with links placed randomly. Shared Targets per Controller (STC) and Pairwise Overlap (PO) measurements are presented as mean over all controllers or pairs of controllers, respectively. Mean and standard deviation of overlap parameters in the shuffled and random networks varied less than 2% over 5 simulations. (DOCX) [file pone.0029374.s014.docx]

**Table S4: Comparison of overlap parameters in biological networks to random.** Shuffled networks have equal numbers of nodes and links, as well as equivalent link distributions, as the biological network. Random networks have equal numbers of nodes and links, with links placed randomly. Shared Targets per Controller (STC) and Pairwise Overlap (PO) measurements are presented as mean over all controllers or pairs of controllers, respectively. Mean and standard deviation of overlap parameters in the shuffled and random networks varied less than 2% over 5 simulations.

| **Shared Targets per Controller (STC)** | | |  |  |  |  |
| --- | --- | --- | --- | --- | --- | --- |
| **Network** | **Biological** | | **Shuffled  (equal link dist.)** | | **Random  (equal # nodes, links)** | |
| *Average over controllers* | *Mean* | *Std dev* | *Mean* | *Std dev* | *Mean* | *Std dev* |
| Human TF | 97.8% | 2.4% | 97.7% | 1.4% | 100.0% | 0.2% |
| Human miRNA | 95.3% | 3.0% | 98.7% | 0.9% | 99.5% | 0.4% |
| Human Kinase | 73.5% | 34.3% | 79.7% | 27.1% | 90.3% | 10.7% |
| Yeast TF | 98.1% | 4.3% | 98.7% | 1.4% | 99.9% | 0.2% |
| Yeast Kinase | 86.0% | 14.3% | 87.5% | 9.9% | 94.8% | 3.2% |
| E. coli TF | 73.5% | 36.1% | 83.4% | 17.3% | 88.9% | 7.5% |
|  |  |  |  |  |  |  |
| **Pairwise shared targets (PO)** | |  |  |  |  |  |
| **Network** | **Biological** | | **Shuffled  (equal link dist.)** | | **Random  (equal # nodes, links)** | |
| *Average over controllers* | *Mean* | *Std dev* | *Mean* | *Std dev* | *Mean* | *Std dev* |
| Human TF | 4.5% | 1.0% | 4.5% | 0.4% | 1.9% | 0.2% |
| Human miRNA | 7.1% | 1.1% | 7.3% | 0.9% | 3.4% | 0.1% |
| Human Kinase | 1.5% | 1.3% | 1.6% | 1.4% | 0.9% | 0.2% |
| Yeast TF | 6.3% | 1.8% | 6.5% | 1.2% | 3.5% | 0.1% |
| Yeast Kinase | 8.5% | 3.7% | 9.0% | 3.2% | 3.4% | 0.3% |
| E. coli TF | 1.1% | 0.9% | 1.5% | 0.7% | 1.3% | 0.2% |
