# Supplementary material for: Statistical Properties and Robustness of Biological Controller-Target Networks
Source: PLoS One. 2012 Jan 3;7(1):e29374. doi: 10.1371/journal.pone.0029374 (PMC3250441; doi:10.1371/journal.pone.0029374)
Supplement: Table S5 — Evaluation of fitting models. Higher R-squared values for each network are in bold. (DOCX) [file pone.0029374.s015.docx]

**Table S5: Evaluation of fitting models**. Higher R-squared values for each network are in bold.

| Controllers per target (incoming) | | |  |  |  |  |
| --- | --- | --- | --- | --- | --- | --- |
| **Exponential fit** |  |  |  | **Scale-free fit** | |  |
| *Network* | *slope* | *R squared* | | *Network* | *slope* | *R squared* |
| Human miRNA | 13.2 | **0.964** |  | Human miRNA | 0.92 | 0.881 |
| Human TF | 10.1 | **0.967** |  | Human TF | 0.88 | 0.867 |
| E. coli TF | 9.2 | **0.953** |  | E. coli TF | 1.49 | 0.91 |
| Yeast TF | 10.4 | **0.997** |  | Yeast TF | 1.01 | 0.753 |
| Human Kinase | 3.9 | 0.916 |  | Human Kinase | 1.37 | **0.939** |
| Yeast Kinase | 3.6 | 0.92 |  | Yeast Kinase | 1.18 | **0.958** |
|  |  |  |  |  |  |  |
| Targets per control (outgoing) | | |  |  |  |  |
| **Exponential fit** |  |  |  | **Scale-free fit** | |  |
| *Network* | *slope* | *R squared* | | *Network* | *slope* | *R squared* |
| Human miRNA | 0.0047 | **0.964** |  | Human miRNA | 0.62 | 0.579 |
| Human TF | 0.028 | **0.74** |  | Human TF | 1.18 | 0.567 |
| E. coli TF | 0.028 | 0.697 |  | E. coli TF | 0.77 | **0.928** |
| Yeast TF | 0.0059 | **0.948** |  | Yeast TF | 0.47 | 0.6055 |
| Human Kinase | 0.15 | 0.837 |  | Human Kinase | 0.78 | **0.911** |
| Yeast Kinase | 0.015 | **0.9646** |  | Yeast Kinase | 0.65 | 0.758 |
